# The impact of extraction method and pollen concentration on community composition for pollen metabarcoding

**DOI:** 10.1002/aps3.11601

**Published:** 2024-08-06

**Authors:** Arne Devriese, Gerrit Peeters, Rein Brys, Hans Jacquemyn

**Affiliations:** ^1^ Department of Biology, Plant Conservation and Population Biology KU Leuven Leuven B‐3001 Belgium; ^2^ Research Institute for Forest and Nature Gaverstraat 4 Geraardsbergen B‐9500 Belgium

**Keywords:** bumblebees, Illumina sequencing, metabarcoding, plant–pollinator interactions, pollen

## Abstract

**Premise:**

Plants and pollinators closely interact with each other to form complex networks of species interactions. Metabarcoding of pollen collections has recently been proposed as an advantageous method for the construction of such networks, but the extent to which diversity and community analyses depend on the extraction method and pollen concentration used remains unclear.

**Methods:**

In this study, we used a dilution series of two pollen mixtures (a mock community and pooled natural pollen loads from bumblebees) to assess the effect of mechanical homogenization and two DNA extraction kits (spin column DNA extraction kit and magnetic bead DNA extraction kit) on the detected pollen richness and community composition.

**Results:**

All species were successfully detected using the three methods, even in the most dilute samples. However, the extraction method had a significant effect on the detected pollen richness and community composition, with simple mechanical homogenization introducing an extraction bias.

**Discussion:**

Our findings suggest that all three methods are effective for detecting plant species in the pollen loads on insects, even in cases of very low pollen loads. However, our results also indicate that extraction methods can have a profound impact on the ability to correctly assess the community composition of the pollen loads on insects. The choice of extraction methodology should therefore be carefully considered to ensure reliable and unbiased results in pollen diversity and community analyses.

Biotic pollination is an ecologically important and well‐studied interaction between plants and animals, especially insects (Ollerton et al., 2011). As plants are seldom visited by a single pollinator (Waser et al., 1996) and most pollinators are polylectic (Waser and Ollerton, 2006), the interactions between plants and pollinators are commonly described as plant–pollinator networks (Jordano, 1987; Memmott, 1999; Bascompte et al., 2003; Vázquez et al., 2009). In general, plant–pollinator networks can be divided into two categories: visitation networks and pollen‐transport networks (Alarcón, 2010). The elucidation of both types of networks is very time consuming and often requires expert knowledge (Bell et al., 2016). Visitation networks are constructed based on field pollinator observations (e.g., Olesen et al., 2008; Pornon et al., 2016), while pollen‐transport networks are traditionally based on the microscopic identification of obtained insect pollen loads (Alarcón, 2010; Jędrzejewska‐Szmek and Zych, 2013). These microscopic identifications are very time consuming and are often characterized by a limited taxonomic resolution. Recently, metabarcoding has been used as an alternative method for identifying the composition of pollen collected from the bodies of pollinators (Vamosi et al., 2017). The main advantage of this method is that it requires less expert knowledge and is less time consuming, allowing for the analysis of a larger number of samples (Sickel et al., 2015; Bell et al., 2016; Leontidou et al., 2018).

Despite the great potential of metabarcoding for the construction of plant–pollinator networks (Pornon et al., 2016; Bell et al., 2017; Galliot et al., 2017; Vamosi et al., 2017; Gous et al., 2019; Macgregor et al., 2019), it has not yet become generally implemented as a standard method in plant–pollinator research (Arstingstall et al., 2021). This is partly because of some methodological hurdles that must be overcome, including the lack of a standardized methodological guideline and the currently limited quantitative capacity of the method resulting from amplification biases (Pawluczyk et al., 2015; Pornon et al., 2016; Bell et al., 2016, 2019). One of the main obstacles for implementing metabarcoding with insect pollinator pollen loads is the low pollen loads carried by many of them, such as syrphid flies, male bees, and lepidopterans, posing a challenge for the collection of a sufficient amount of DNA (Gyan and Woodell, 1987; Lucas et al., 2018; Macgregor et al., 2019). Nevertheless, many of these insects can still be effective pollinators (Ballantyne et al., 2015), and the collection and efficient DNA extraction of their pollen loads are therefore important for the construction of biologically relevant networks via metabarcoding (Bell et al., 2016). As there may be little room for improving pollen collection, the optimization of downstream processes, including DNA extraction, is needed (Swenson and Gemeinholzer, 2021).

Information about protocol optimization for the maximal DNA recovery of small pollen loads is limited. Swenson and Gemeinholzer (2021) focused specifically on the exine rupture of pollen, showing that increased exine rupture increased the obtained DNA quantity, but overprocessing the samples had a detrimental effect on DNA quality. Leontidou et al. (2018) compared different DNA extraction methods for metabarcoding airborne pollen, which typically involve pollen samples that are much larger and free from pollinator‐specific contaminants, such as setae from the pollinator's body. In most cases, research on this topic relies on preliminary tests to select an appropriate protocol, but the detailed results are rarely completely published (Bell et al., 2019; Gous et al., 2019; but see Suchan et al., 2019). Additionally, most research circumvents the problem of low pollen loads by focusing on pollinator groups typically carrying high pollen loads, like honey bees and bumblebees (Cornman et al., 2015; Richardson et al., 2015; De Vere et al., 2017; Smart et al., 2017; Potter et al., 2019; Leidenfrost et al., 2020). Even research targeting syrphid flies mainly focuses on the genus *Eristalis*, which comprises typically large and hairy species that can carry high pollen loads (Lucas et al., 2018).

At present, three main categories of protocols are employed to prepare pollen samples for amplification using PCR. First, given that pollen samples are typically relatively pure with limited soluble contaminants, DNA extraction may not always be necessary, although in some cases amplification by PCR is possible after only mechanically disrupting the samples (Suchan et al., 2019). Because DNA extraction is not required, this method stands out as the most time‐ and cost‐effective; however, the disruption of pollen material varies between species, with some releasing their genetic material more easily than others (Swenson and Gemeinholzer, 2021), potentially resulting in biased communities. An alternative protocol involves a cetyltrimethylammonium bromide (CTAB) DNA extraction (Cornman et al., 2015), which requires the exine layer of the pollen to be disrupted through mechanical homogenization, after which a lysis buffer, which can be customized for the specific sample properties, is added to disrupt the cell membrane. The resulting lysate is then washed several times with solvents before the DNA is precipitated out of the aqueous solution using ethanol. The obtained DNA pellet is also subsequently washed several times. To further purify the DNA extract, RNase and proteinase K can be used to remove RNA and proteins, respectively, from the sample. While this method generates pure and high‐quality DNA, even from degraded samples or samples with a high amount of contaminants, it demands specific handling skills and is a time‐consuming process, often spanning two days for a single extraction. Additional disadvantages include the use of harmful chemicals such as chloroform and, in some protocols, phenol (Porebski et al., 1997). Additionally, separating insoluble insect‐derived impurities from the DNA can be challenging with this method as chitin can rarely be fully digested and may contaminate the DNA pellet during the ethanol precipitation (Campos and Gilbert, 2019).

The third category involves the use of commercially available DNA extraction kits that rely on a solid agent for DNA binding rather than ethanol precipitation (Kraaijeveld et al., 2015; Pornon et al., 2016; Bell et al., 2017; Gous et al., 2019; Swenson and Gemeinholzer, 2021). In this approach, the samples are initially mechanically homogenized, followed by a chemical lysis step. Subsequently, the DNA is bound to a solid agent, which is then washed several times to remove impurities. Finally, the DNA is redissolved in an elution buffer. These kits require less intricate handling skills, are less time consuming, and involve fewer harmful chemicals. They are, on the other hand, more expensive and may have limits regarding the amount of DNA they can purify. Within this category, there are two main methods. The most common uses extraction kits that rely on spin column extractions that use resin filters to bind the DNA while the sample is centrifuged (Kraaijeveld et al., 2015; Pornon et al., 2016; Bell et al., 2017; Gous et al., 2019; Swenson and Gemeinholzer, 2021). The other approach is the use of magnetic bead kits, which have recently become more commonly used for pollen extraction (Leontidou et al., 2018). These kits use beads that are mixed with the sample to bind the DNA, with separation achieved using a magnetic field. Although the quality of the DNA sample strongly affects all downstream steps, the impact of DNA extraction techniques on the community representation of pollen collections and their efficacy for small samples has not been studied in detail.

In this study, we (i) investigated the effect of mechanical homogenization and two DNA extraction methods (spin column DNA extraction kit and magnetic bead DNA extraction kit) on the taxonomic representation of mixed pollen samples, and (ii) assessed their effectiveness for metabarcoding at low pollen concentrations. All experiments were conducted with artificial pollen communities and pollen collected from the bodies of bumblebees. For both pollen communities, a five‐fold serial dilution was used and all analyses were performed using the internal transcribed spacer 2 nuclear ribosomal fragment (ITS2).

## METHODS

### Pollen collection

Two different pollen sources were used to perform the analyses. First, a mock community was constructed with pollen directly collected from the flowers of 12 different plant species growing in a dune slack in the Ter Yde nature reserve (Koksijde, Belgium) (Table 1). The flowers were harvested just before anthesis and brought to the laboratory, where the anthers were removed with sterile tweezers and dried in paper bags. After drying, the pollen was shaken loose from the anthers and stored in 100% ethanol at −20°C. All species in the mock community have tricolpate pollen, except *Epipactis palustris* (L.) Crantz, which has ulcerate pollen. Most species produce small pollen grains, with the longest polar axis in equatorial view varying between 16 and 20 µm (Table 1). *Rhinanthus angustifolius* C. C. Gmel produces the largest pollen, with the longest polar axis in equatorial view around 31–35 µm (Table 1). For the second pollen mixture, bumblebees were caught by manual netting at the same time as the flower harvesting and in the same dune slacks, and were then stored dry at −20°C. Pollen was collected from 11 bumblebee individuals by removing the pollen clumps from the corbiculae with a spatula.

**Table 1:** Species composition of the mock community used in this study, with shape, size (longest polar axes), indication of the intended concentrations per species, and their respective proportions. The shape and size of the pollen was retrieved from PalDat (https://www.paldat.org).

Species	Pollen shape	Longest polar axis (µm)	Intended concentration (pollen grains/mL)	Proportion of the community composition
*Lotus corniculatus*	Tricolporate	16–20	120,000	0.477
*Parnassia palustris*	Tricolporate	16–20	40,000	0.159
*Centaurium erythraea*	Tricolporate	26–30	16,000	0.0636
*Pyrola rotundifolia*	Tetrad, tricolporate	NA	16,000	0.0636
*Hypericum perforatum*	Tricolporate	16–20	16,000	0.0636
*Epipactis palustris*	Pollinium, ulcerate	NA	14,500	0.0576
*Blackstonia perfoliata*	Tricolporate	16–20	14,000	0.0556
*Centaurium littorale*	Tricolporate	26–30	3200	0.0127
*Hypericum tetrapterum*	Tricolporate	16–20	3200	0.0127
*Rhinanthus angustifolius*	Tricolporate	31–35	3200	0.0127
*Ononis repens*	Tricolporate	21–25	3200	0.0127
*Trifolium repens*	Tricolporate	21–25	2400	0.00954

*Note*: NA = not available.

### Sample preparation

Pollen suspensions were prepared in a 2:1 ethanol:glycerol solution (Bell et al., 2019). Both suspensions were created with a target concentration of 2.5 × 10^5^ pollen grains/mL. This concentration was chosen so that one 0.5 mL sample corresponds to the number of pollen grains retrieved from the corbiculae of one bumblebee. First, the artificial pollen mixture for the mock community was created by removing the ethanol in which the pollen was stored and replacing it with the ethanol:glycerol mixture. Subsequently, pollen concentrations were determined using a Neubauer improved counting chamber (0.0025 mm² grid, 0.100 mm depth) (Paul Marienfeld, Lauda‐Königshofen, Germany). Next, proportions were chosen at which pollen from each species was added to the mock community to create a gradient of relative abundances (Table 1). For the second suspension, the collected pollen clumps were pooled and manually broken up with a spatula, suspended in the ethanol:glycerol mixture, and vortexed to achieve a homogeneous suspension. The pollen concentrations were determined using a Neubauer improved counting chamber, then diluted to the desired concentration to ensure that both stock solutions had equal total pollen concentrations. Using these stock suspensions, two five‐fold dilution series with four dilution steps were made. For each stock, this resulted in five pollen suspensions: undiluted, five‐fold diluted, 25‐fold diluted, 125‐fold diluted, and 625‐fold diluted. For all pollen suspensions, the actual pollen concentration was determined using a Neubauer improved counting chamber. These concentrations were used in all subsequent analyses.

### DNA extraction

DNA samples for PCR amplification were prepared using three methods: (1) mechanical homogenization without extraction, hereafter referred to as the “direct method,” (2) spin column DNA extraction (Plant/Fungi DNA Isolation Kit; Norgen Biotek, Thorold, Ontario, Canada), and (3) magnetic bead DNA extraction (Plant DNA Isolation Kit Magnetic Bead System; Norgen Biotek). For each method, three replicates were made per pollen suspension, yielding a total of two pollen mixtures × five dilutions × three extraction methods × three replicates = 90 samples. The ethanol and glycerol mixture was removed by centrifuging at 14,000 rpm for 15 min then decanting the supernatant.

For the direct method, the pollen was resuspended in 25 µL elution buffer and homogenized in a bead mill homogenizer with two 3‐mm and six 2‐mm glass beads, which was shaken at 4 m/s for seven 30‐s cycles. Subsequently, 75 µL of elution buffer was added and centrifuged at 14,000 rpm for 5 min, after which the elution buffer was pipetted off into a new 1.5‐mL microtube, resulting in a 100‐µL eluate.

In both the spin column DNA extraction and magnetic bead DNA extraction processes, pollen samples were first resuspended in 250 µL lysis buffer for chemical lysis. Subsequently, the samples were homogenized in a bead mill homogenizer using two 3‐mm and six 2‐mm glass beads and shaken at 6.95 m/s for two 30‐s cycles. Because of the larger volume of elution buffer, a higher speed than the direct method was achievable without damaging the sample tubes. Afterwards, an additional 250 µL lysis buffer was added, after which the protocols were executed according to the manufacturer's instructions for both DNA extraction kits (Norgen Biotek). For the spin column DNA extraction, an elution volume of 50 µL was used for the most highly concentrated samples, while 30 µL was used for the diluted samples. For the magnetic bead DNA extraction, an elution volume of 75 µL was used. This increased volume was necessary to facilitate pipetting off the elution buffer without any interference from the magnetic beads.

### PCR and sequencing

The PCR was performed using the primer pair ITS2‐S2F/ITS4R (White et al., 1990; Chen et al., 2010), optimized for dual indexing on an Illumina MiSeq (Illumina, San Diego, California, USA). The reaction was performed using the ALLin Mega HiFi Red Mastermix (highQu, Kraichtal, Germany) with an initial denaturation at 95°C for 60 s, followed by 30 cycles of denaturation at 99°C, annealing at 50°C, and extension at 72°C of 15 s each. Subsequently, the PCR product was purified using AMPure XP magnetic beads (Beckman Coulter, Brea, California, USA) in a bead:sample ratio of 1.8:1. Illumina sequencing was performed at Genomics Core Leuven, Leuven, Belgium.

### Bioinformatics

Forward and reverse paired‐end reads were received as de‐multiplexed FASTQ files. Paired‐end reads were merged and filtered using USEARCH (Edgar, 2010). A minimum length of 200 bp was set, and paired‐end reads with a total expected error higher than 1.0 were discarded. Zero‐radius operational taxonomic units (ZOTUs) were identified using the unoise3 algorithm with the default settings from USEARCH (Edgar, 2013). Taxonomy was assigned using our own reference database (Appendix S1) and the classify‐consensus‐vsearch algorithm implemented in QIIME2 (Rognes et al., 2016; Bolyen et al., 2019). To compensate for the potential incompleteness of our reference database, unassigned ZOTUs were manually assigned using a BLAST search in the National Center for Biotechnology Information (NCBI) GenBank (https://blast.ncbi.nlm.nih.gov/Blast.cgi). As a final filtering step to eliminate false reads, taxa that were observed fewer than three times in at least 20% of the samples, as well as taxa with a relative frequency below 0.5%, were removed using the phyloseq package in R version 4.0.3 (McMurdie and Holmes, 2013; R Core Team, 2014). This threshold was selected based on the work of Drake et al. (2022). Although a threshold based on a receiver operating characteristic (ROC) approach as described by Tommasi et al. (2021) was considered, we deemed this approach overly stringent, leading to a high rate of false negatives.

### Data analysis

A species–sample data matrix was generated by aggregating the read counts of all ZOTUs assigned to the same species. To test whether the observed community composition was affected by the extraction method or dilution, a permutational multivariate analysis of variance (PERMANOVA) was performed separately for each pollen mixture, using the Bray–Curtis coefficient as the distance measure and extraction method and dilution as independent variables. The adonis2 function from the R package Vegan (Oksanen et al., 2022) was used for this purpose. Finally, as metabarcoding is not quantitative, the effects of extraction method and dilution on the observed species richness were tested separately for each pollen mixture using an analysis of covariance (ANCOVA). In this analysis, species richness served as the dependent variable, while extraction method and dilution were used as independent variables. This was performed in R version 4.0.3 using the package lme4 (Bates et al., 2015). To perform pairwise comparisons of the species richness between dilutions, Tukey's test was applied using the lsmeans function from the emmeans package (Lenth, 2023).

## RESULTS

**Figure 1:**
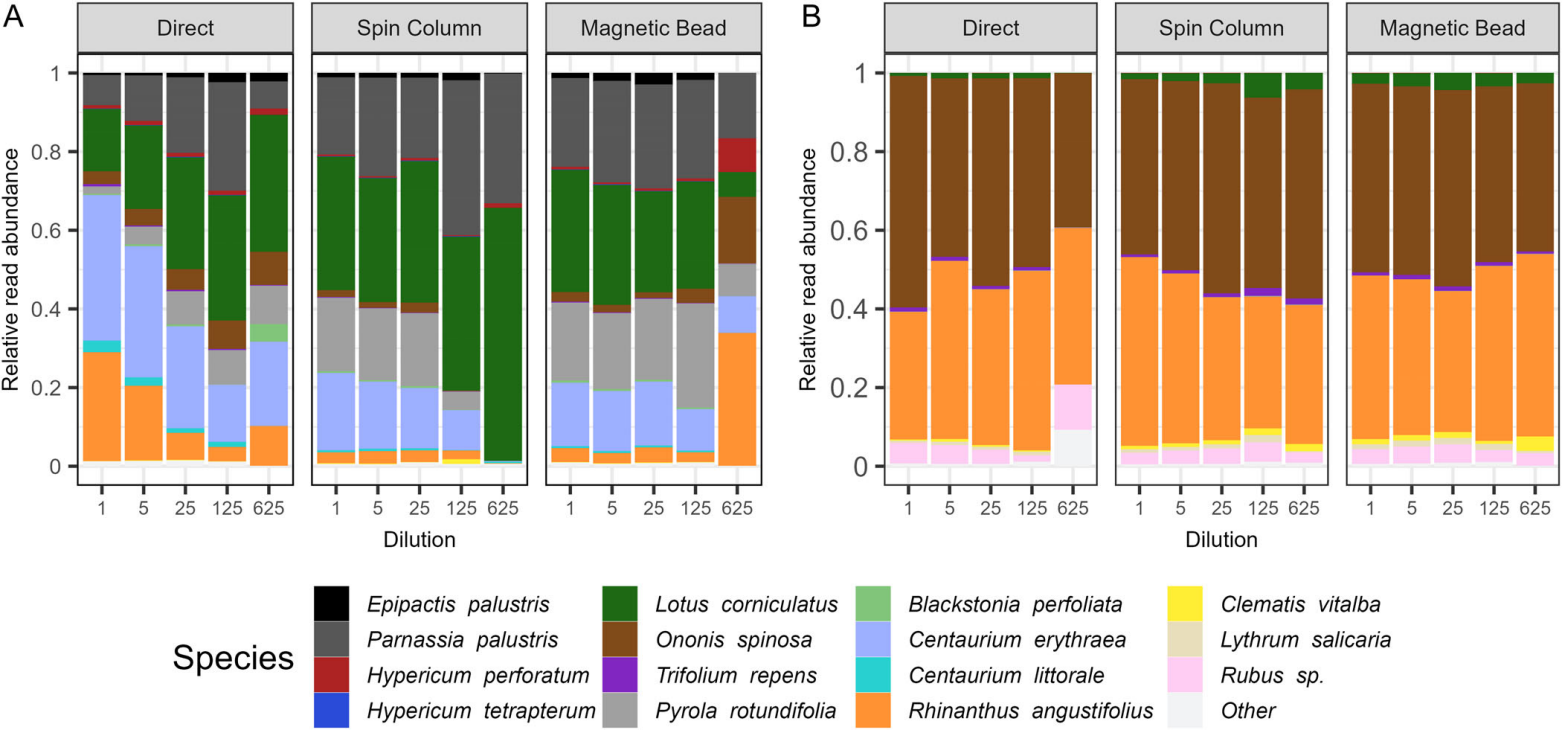
Relative read abundance (proportions of reads detected for each species) in the mock community samples (A) and bumblebee samples (B) for each dilution step, shown for three different extraction methods. Species are ordered based on their expected proportion per sample in the mock community.

The sequencing of the mock community yielded 1,326,198 reads, which were assigned to 335 ZOTUs. After filtering, 1,314,504 reads (99.1%) and 41 ZOTUs remained. These were assigned to 13 species (all 12 original species and *Clematis vitalba* L.) (Figure 1, Table 2). Two species, *Lotus corniculatus* L. and *Parnassia palustris* L., were assigned to noticeably more ZOTUs than the other species. The bumblebee community yielded 1,449,138 reads, which were assigned to 300 ZOTUs. After filtering, 1,437,534 reads (99.2%) and 44 ZOTUs remained, which were assigned to 16 species (Table 2). Again, *Lotus corniculatus* was assigned to more ZOTUs than most other species.

**Table 2:** Number of detected zero‐radius operational taxonomic units (ZOTUs) per species and the median number of reads per species.

	Mock community		Bumblebee pollen samples	
Species	No. of ZOTUs	Median total reads	No. of ZOTUs	Median total reads
*Lotus corniculatus*	8	9430	7	648
*Parnassia palustris*	8	6841	0	–
*Centaurium erythraea*	4	5246	0	–
*Pyrola rotundifolia*	2	2986	1	16
*Hypericum perforatum*	1	196	1	3
*Epipactis palustris*	1	349	0	–
*Blackstonia perfoliata*	2	123	0	–
*Centaurium littorale*	1	195	0	–
*Hypericum tetrapterum*	1	40	0	–
*Rhinanthus angustifolius*	3	1023	5	13,396
*Ononis repens*	2	735	4	16,320
*Trifolium repens*	3	36	3	296
*Clematis vitalba*	1	0	1	267
*Hypericum* sp.	2	258	1	11
*Lythrum salicaria*	0	–	1	353
*Rubus* sp.	0	–	6	1270
*Anchusa officinalis*	0	–	1	59
*Galium verum*	0	–	1	0
*Eupatorium cannabinum*	0	–	1	1
*Centaurea jacea*	0	–	5	36
*Hieracium umbellatum*	0	–	1	12
*Hypochaeris radicata*	0	–	1	3
*Leontodon saxatilis*	0	–	1	2
*Sinapis arvensis*	0	–	1	15
Unassigned	2	5	2	25

**Figure 2:**
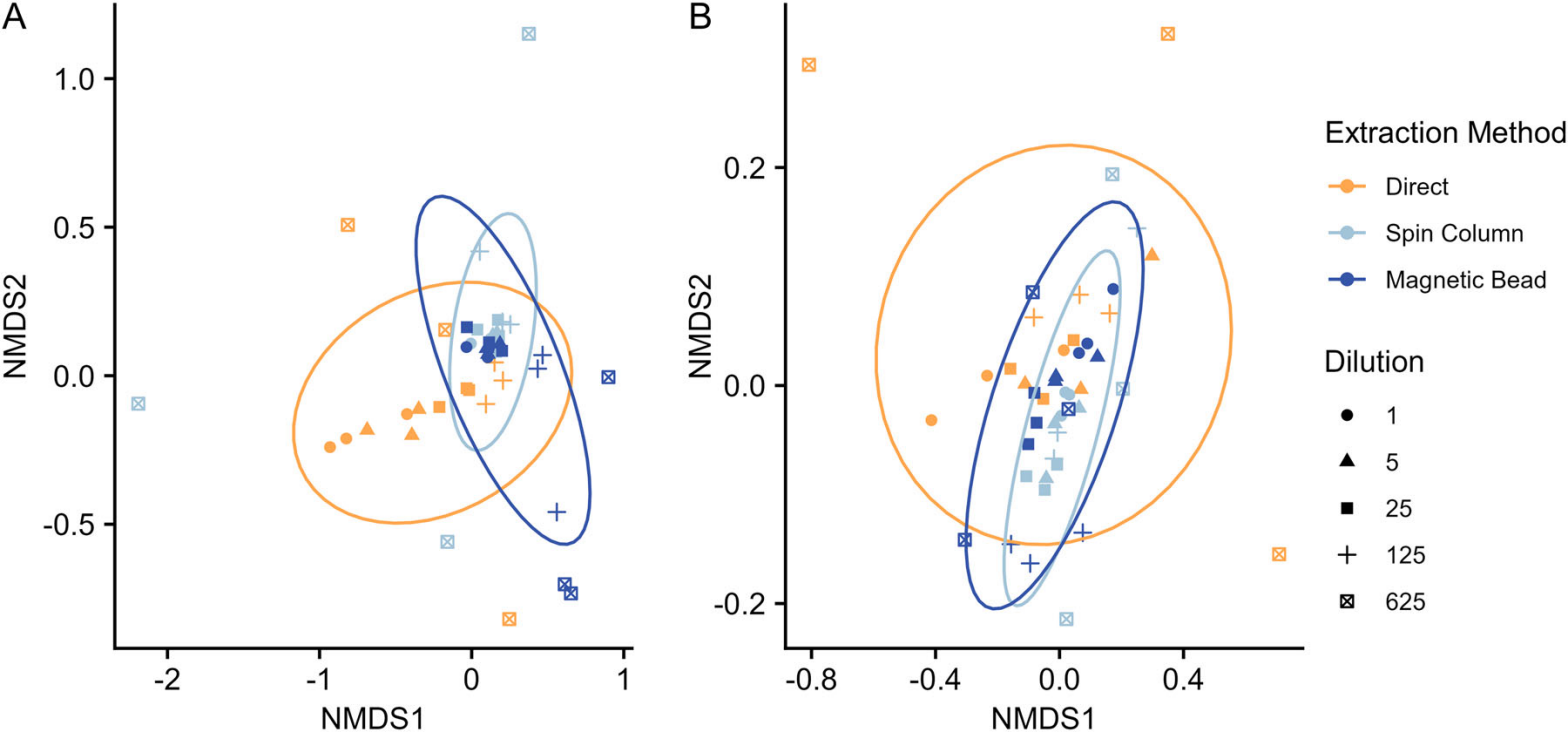
Non‐metric multidimensional scaling (NMDS) of the mock community pollen assemblage (A) and bumblebee pollen assemblage (B). Ellipses indicate 95% confidence level.

The extraction method significantly affected the community composition of the mock community (*R* ^2^ = 0.23, *F*_2,44_ = 6.87, *P* < 0.001), but not of the bumblebee samples (*R* ^2^ = 0.08, *F*_2,44_ = 1.72, *P* = 0.13) (Figure 2A). Samples from the direct method with high pollen concentrations clearly diverge from the other samples containing high pollen concentrations. When compared with samples with lower concentrations or samples obtained using an extraction kit, *Rhinanthus angustifolius*, *Centaurium erythraea* Rafn, and to a lesser extent *Centaurium littorale* (Turner) Gilmour and *Ononis spinosa* L. had a higher relative read abundance, while *Pyrola rotundifolia* L. and to a lesser extent *Lotus corniculatus* and *Parnassia palustris* had a lower relative read abundance (Figure 1A). Extraction method did not have a significant effect on the detected species richness (mock community: χ^2^ = 0.40, *P* = 0.82; bumblebee pollen load: χ^2^ = 5.62, *P* = 0.06).

Between most concentrations, the species composition of the pollen communities was consistent both for the mock community and the bumblebee pollen loads, with only the most diluted samples strongly diverging (Figure 1). The PERMANOVA showed that dilution had a significant effect on the observed pollen community composition of the mock community (Figure 2A), but not for the bumblebee pollen loads (Figure 2B) (mock community: *R* ^2^ = 0.15, *F*_4,44_ = 2.25, *P* = 0.005; bumblebee pollen loads: *R* ^2^ = 0.05, *F*_4,44_ = 0.59, *P* = 0.81). The detected species richness was significantly affected by the dilution of both pollen mixtures (mock community: χ^2^ = 51.63, *P* < 0.001; bumblebee pollen load: χ^2^ = 75.67, *P* < 0.001); however, only the most dilute samples showed significantly lower species richness than the other dilutions (Appendix S2).

## DISCUSSION

Most insects collect pollen from several plant species (Waser et al., 1996). Accurate detection and identification of plant species in insect pollen collections are crucial for reconstructing the architecture of the plant–pollinator networks in natural environments (Ballantyne et al., 2015; Bell et al., 2017) and assessing the diversity of plant species visited by insects to meet their nutritional demands (Leidenfrost et al., 2020; Verbeke et al., 2023). DNA metabarcoding is a popular tool for assessing diversity in insect pollen collections, but methodological insights regarding optimal DNA extraction and amplification protocols, sequencing, and data analysis are still lacking (Pawluczyk et al., 2015; Pornon et al., 2016; Bell et al., 2016, 2019; Arstingstall et al., 2021). In this study, we investigated the impact of different extraction methods and pollen concentrations on the detected community composition of pollen mixes.

We observed that the direct extraction method, without chemical lysis agents, caused a bias towards *Rhinanthus*, *Centaurium*, and *Ononis*. These species, like most others in the mock community, have tricolporate pollen; thus, this bias could likely be attributed to the larger size of their pollen grains being more susceptible to mechanical disruption (Gibbons et al., 2014). By contrast, *Pyrola rotundifolia*, which was less abundantly detected, possesses pollen organized in tetrads, which may be more resistant to mechanical disruption. Our findings are consistent with those of Swenson and Gemeinholzer (2021), who reported similar discrepancies in species detection using different extraction methods. Our results further underscore the importance of using chemical lysis agents to disrupt the cell membrane and allow the release of DNA from pollen grains that were not mechanically disrupted. Despite this, the influence of extraction bias is often overlooked when assessing the (semi‐)quantitative capabilities of metabarcoding.

As there was no clear difference in the community composition or species detection between the two commercially available extraction kits, the choice of the most appropriate extraction method might depend on factors such as ease of use and equipment availability. The magnetic bead extraction protocol involves more meticulous pipetting steps, both at the stage of adding the magnetic beads and the removal of eluent, and thus requires more skills than the spin column method. However, this method is the only one compatible with robotics, allowing for the automated processing of a large number of samples and mitigating its time constraints.

Even though pollen concentration had a significant effect on community composition and species richness, our results are encouraging. Notably, community composition only started to change in the second most diluted samples, which had pollen loads of around 3000 to 4000 pollen grains. These loads are considerably lower than the pollen loads reported on the bodies of bees and flies by Cullen et al. (2021). Furthermore, species richness was significantly reduced only in the most diluted samples. The undetected plants in these samples were rare species that could have been absent in samples due to stochastic effects. This likely explains why, in the most dilute samples of the mock community, the species constituting only 1% of the total pollen load (*Hypericum tetrapterum* Fr., *Centaurium littorale*, and *Trifolium repens* L.) were detected in some but not all of these samples. The ecological relevance of such rare links in a plant–pollinator network may also be questioned, given the low probability that rarely occurring pollen will be deposited on a conspecific stigma.

In addition, we observed no discernible effects of the extraction method or dilution level on the detected community composition of the bumblebee pollen loads. This is likely attributable to the strong dominance of species such as *Rhinanthus angustifolius* and *Ononis spinosa* in the pollen loads. The pollen from both species was easily disrupted by bead milling, resulting in no extraction bias. This suggests that the presence or absence of bias depends on the species composition of the pollen sample. Furthermore, the prevalence of these two dominant species most likely masked any variations in the frequency of the rare species. This explains the significant decrease in species richness in the most dilute samples, despite the absence of significant changes in community composition. Here again, the absence of rare species might be attributed to stochastic effects and their ecological relevance can be questioned.

Finally, even though we did not observe significant effects of the extraction method on species richness, there was a trend toward lower species richness in the bumblebee pollen loads when the direct method was used. However, when a slightly more strict filter was applied, requiring a minimal relative frequency of 0.6% for a taxon to be retained, one fewer species was detected in the bumblebee pollen loads. With this different filter, there was a significant effect of extraction method on the detected species richness (χ^2^ = 6.18, *P* = 0.046). This implies that the methodology used to process insect pollen loads can affect the detected species richness.

## CONCLUSIONS

We conclude that all three tested methods are viable for retrieving DNA from pollen loads on insect bodies, even when dealing with low pollen quantities. While mechanical homogenization without the DNA extraction of pollen samples is the most cost‐effective option, its results are contingent upon sample composition and may introduce a significant bias. Additionally, our results indicated that this method could result in a lower detected species richness. Consequently, it is not a suitable choice if the aim of the study is to reveal differences in species abundances or to perform more detailed quantitative analyses. For studies seeking consistency and avoiding biases, we recommend the use of commercially available DNA extraction kits. These kits offer more reliable results, making them preferable for rigorous and quantitative analyses.

## AUTHOR CONTRIBUTIONS

A.D. and R.B. jointly conceived the research idea. The optimization of the protocol and further lab work were performed by A.D. and G.P. The data analysis was conducted by A.D., with input from H.J. The manuscript was primarily written by A.D., and all authors provided input and approved the final manuscript.

## Supporting information


**Appendix S1**. Construction of the reference database for metatabarcoding.

**Appendix S2**. Correlation coefficients, test statistics, and *P* values from the Tukey's tests between the observed species richness in the different dilution steps.

## Data Availability

Raw sequencing data are available at: https://www.ncbi.nlm.nih.gov/sra/PRJNA1034601.
