# Supplementary material for: The impact of extraction method and pollen concentration on community composition for pollen metabarcoding
Source: Appl Plant Sci. 2024 Aug 6;12(5):e11601. doi: 10.1002/aps3.11601 (PMC11443440; doi:10.1002/aps3.11601)
Supplement: Supplementary file 1 — Appendix S1. Construction of the reference database for metatabarcoding. [file APS3-12-e11601-s002.docx]

**Appendix S1.** Construction of the reference database for metatabarcoding.

**Sample collection**

In the summer of 2022, leaf samples were collected from multiple dune areas along the Belgian coast and stored dry on silica gel. The list of collected species was based on prior vegetation surveys of the same areas. Any additional species that were observed were collected as well.

**DNA extraction**

DNA was extracted by homogenizing 15–18 mg of the dried sample in a bead mill homogenizer with two 3-mm and six 2-mm glass beads and shaken at 6.95 m/s for two 30-s cycles with 250 µL lysis buffer. Subsequently, another 250 µL lysis buffer was added to the samples, which were then extracted using a Plant/Fungi 96-Well Plate Kit (Norgen Biotek, Thorold, Ontario, Canada), following the manufacturer’s protocol.

**PCR and sequencing**

The PCR was performed using ALLIn HS Red Taq Mastermix (highQu, Kraichtal, Germany) and the *ITS2* primers ITS2-S2F/ITS4R (White et al., 1990; Chen et al., 2010). The initial denaturation was performed at 95°C for 2 min, followed by 40 cycles of denaturation at 95°C, annealing at 60°C, and extension at 72°C of 15 s each. The PCR product was purified using AMPure XP magnetic beads (Beckman Coulter, Brea, California, USA) in a bead:sample ratio of 1.8:1. Sanger sequencing was performed by Genomics Core Leuven, Leuven, Belgium. The quality of each run was visually checked on 1.5% agarose gel. For species with a poor-quality sequence in both the forward and reverse runs, new samples were extracted and sequenced. Consensus sequences were obtained by aligning and assembling the forward and reverse runs in Geneious Prime, using the Geneious alignment (Geneious Prime 2021.0.1). These consensus sequences were used as reference sequences for the pollen analysis.

**REFERENCES**

Chen, S., H. Yao, J. Han, C. Liu, J. Song, L. Shi, Y. Zhu, et al. 2010. Validation of the ITS2 region as a novel DNA barcode for identifying medicinal plant species. *PLoS ONE* 5(1): e8613. https://doi.org/10.1371/JOURNAL.PONE.0008613

White, T. J., T. Bruns, S. Lee, and J. Taylor. 1990. Amplification and direct sequencing of fungal ribosomal RNA genes for phylogenetics. *In* M. A. Innis, D. H. Gelfand, J. J. Sninsky, and T. J. White [eds.], PCR Protocols: A guide to methods and applications, 315–322. Academic Press, Cambridge, Massachusetts, USA. https://doi.org/10.1016/B978-0-12-372180-8.50042-1
