# Supplementary material for: The impact of extraction method and pollen concentration on community composition for pollen metabarcoding
Source: Appl Plant Sci. 2024 Aug 6;12(5):e11601. doi: 10.1002/aps3.11601 (PMC11443440; doi:10.1002/aps3.11601)
Supplement: Supplementary file 2 — Appendix S2. Correlation coefficients, test statistics, and P values from the Tukey's tests between the observed species richness in the different dilution steps. [file APS3-12-e11601-s001.docx]

**Appendix S2.** Correlation coefficients, test statistics, and *P* values from the Tukey’s tests between the observed species richness in the different dilution steps. Only the results of the two strongest dilution steps are shown, as there were no significant effects between the less diluted samples.

| **Comparisons of dilutions** | | | | |
| --- | --- | --- | --- | --- |
| 625-fold vs. | One-fold | Five-fold | 25-fold | 125-fold |
| Mock community | *z* = 5.85  *P* < 0.0001*** | *z* = 5.80  *P* < 0.0001*** | *z* = 5.53  *P* < 0.0001*** | *z* = 4.61  *P* < 0.0001*** |
| Bumblebee pollen samples | *z* = 6.59  *P* < 0.0001*** | *z* = 6.91  *P* < 0.0001*** | *z* = 6.91  *P* < 0.0001*** | *z* = 4.80  *P* < 0.0001*** |
